# A Critical Examination of the Practical Implications Derived from the Food Addiction Concept

**DOI:** 10.1007/s13679-019-0326-2

**Published:** 2019-01-31

**Authors:** Adrian Meule

**Affiliations:** 1 Department of Psychology, University of Salzburg, Hellbrunner Straße 34, 5020 Salzburg, Austria; 2 Center for Cognitive Neuroscience, University of Salzburg, Salzburg, Austria

**Keywords:** Obesity, Eating disorders, Binge eating, Substance use disorders, Prevention, Treatment

## Abstract

**Purpose of Review:**

Adopting an addiction perspective on eating disorders and obesity may have practical implications for diagnostic classification, prevention, and treatment of these disorders. The present article critically examines these implications derived from the food addiction concept.

**Recent Findings:**

Introducing food addiction as a new disorder in diagnostic classification system seems redundant as most individuals with an addiction-like eating behavior are already covered by established eating disorder diagnoses. Food addiction may be a useful metaphor in the treatment of binge eating, but would be inappropriate for the majority of obese individuals. Implying an addiction to certain foods is not necessary when applying certain approaches inspired by the addiction field for prevention and treatment of obesity. The usefulness of abstinence models in the treatment of eating disorders and obesity needs to be rigorously tested in future studies.

**Summary:**

Some practical implications derived from the food addiction concept provide promising avenues for future research (e.g., using an addiction framework in the treatment of binge eating or applying abstinence models). For others, however, the necessity of implying an addiction to some foods needs to be scrutinized.

## Introduction

Throughout the past century and in recent years, researchers and clinicians have lively debated whether people can become addicted to certain foods [1]. The current state of affairs can be summarized by distinguishing three prevailing views:

1. Certain foods—usually high-caloric and highly processed foods that contain a large amount of carbohydrates and/or fat—have an addictive potential. Therefore, so-called food addiction represents a substance use disorder [2–4].
2. Other substances of abuse contain a clear addictive agent (e.g., ethanol in alcoholic beverages, nicotine in tobacco, tetrahydrocannabinol in marijuana), but such a specific, addictive substance has not been identified in foods. Therefore, so-called eating addiction represents a non-substance-related, behavioral addiction [5, 6].
3. Neither food nor eating addiction represent valid concepts and—even if they are—they are not necessary [7–9].

Most writings on this topic clearly take up one of the three positions and, thus, it appears that this debate cannot be resolved anytime soon. In line with this, Lacroix and colleagues [10] have recently noted that “the topic of addictive-like eating has evoked polarized positions [and] interpretation of the existing evidence regarding addictive-like eating appears to be driven in part by one’s a priori views” (p. 291). One of few other recent examples that provide a more balanced discussion and suggests tangible future directions is an article by Fletcher and Kenny [11•].

Whether agreeing that the food addiction concept is valid or not, it seems widely accepted that adopting an addiction perspective on food and eating has practical implications for the prevention and treatment of eating disorders and obesity. Yet, what these actually are exactly is often not explicitly spelled out [12] or they are rarely scrutinized dispassionately [10]. Therefore, the present article provides a brief overview of some implications suggested in the literature and aims to evaluate the appropriateness of these suggestions.

## Diagnostic Classification Systems

It stands to reason that if the food or eating addiction concept is accepted as valid, this would imply that food or eating addiction may need to be included as a new addictive disorder in diagnostic classification systems such as the substance-related and addictive disorder section in the fifth revision of the Diagnostic and Statistical Manual of Mental Disorders (DSM-5). However, as has been discussed previously [9, 13], this may be unwarranted if food or eating addiction is not sufficiently distinct from existing diagnostic entities.

Addiction-like eating behavior strongly overlaps with binge eating as displayed by individuals with bulimia nervosa (BN) and binge eating disorder (BED). In a seminal study by Cassin and von Ranson [14], 92% of individuals with BED also met substance dependence criteria that referred to *binge eating* instead of *substance* in a structured clinical interview. When examining findings with the Yale Food Addiction Scale (YFAS) [15] and YFAS 2.0 [16•], prevalence rates varied largely across studies with approximately 40–80% of individuals with BED classified as food addicted [17–20]. In individuals with BN, more than 80% received a food addiction classification across several studies [19–22].

Although these findings indicate a large overlap between food addiction and binge eating-related disorders, there are of course individuals that show addiction-like eating but do not receive a BN or BED diagnosis. In a study by Ivezaj and colleagues [23•], for example, 19% of overweight and obese participants were classified as food addicted according to the YFAS, but did not meet criteria for BED. This implies that there may be indeed a subgroup of overweight and obese individuals that show a clinically significant, disordered eating behavior, but do not receive an established eating disorder diagnosis and, thus, may not receive proper treatment. However, a closer inspection reveals that those with food addiction but without BED did indeed exhibit regular binge eating (*M* = 4.4 objective binge episodes in the past 28 days), but without fulfilling the full criteria for BED. Moreover, interpretation of the study’s findings is limited by the fact that BED classification was based on self-reported binge eating episodes. Thus, it may be that most participants with food addiction but without BED would have received a diagnosis of BED of low frequency and/or limited duration according to DSM-5 in a structured clinical interview. This interpretation is in line with a recent study in which 67% of individuals with a binge/purge-type other specified feeding or eating disorder diagnosis received a food addiction classification with the YFAS 2.0 [24].

In conclusion, it appears that there are some individuals who show a clinically relevant, addiction-like eating behavior but do not receive an established eating disorder diagnosis. However, the distinctiveness between food addiction and established eating disorder diagnoses needs to be rigorously tested in future studies. When employing structured clinical interviews that cover all eating disorders that are included in DSM-5, it may indeed be that the large majority of individuals who receive a food addiction classification would also receive an established eating disorder diagnosis (including those listed in the other specified feeding or eating disorder section in DSM-5). If this is the case, this would make an inclusion of a possible new “food use disorder” in the substance-related and addictive behavior section redundant.

## Obesity Prevention Approaches

It has been suggested that the food addiction concept has implications for the prevention of obesity [25]. Specifically, if certain foods have an addictive potential, policy regulations may be implemented to limit advertising, increase the price of, or restrict access to such foods, similar to alcohol and tobacco regulations [26]. The downside of such restrictive prevention approaches is that they receive low acceptance by the general public who in turn favor less intrusive approaches (e.g., food labeling or public health campaigns) [27]. This is where the food addiction concept may be helpful because support for obesity policies increases when obesity is attributed to environmental forces and decreases when obesity is viewed as stemming from poor personal choices [26, 28].

A shortcoming of this line of reasoning is that only a minority of obese individuals exhibit an addiction-like eating behavior. Studies using the YFAS or YFAS 2.0 found that between approximately 15 and 25% of obese individuals can be classified as food addicted [29–31] (although rates can reach up to 50% in treatment-seeking, extremely obese samples [32]). Accordingly, it has been argued that the food addiction idea is inappropriate for justifying prevention efforts for obesity and that it may even backfire as the food industry may present food addiction as a rare disorder that does not warrant policy changes to influence the general public’s eating [33, 34].

Moreover, although food policy may be inspired by alcohol and tobacco policies, this does necessitate the presence of food addiction. As an example, taxation of sugar-sweetened beverages has been demanded some time ago [35] and has been implemented in some countries in recent years. Indeed, taxation of sugary drinks seems to be associated with a reduction of sugary drink consumption [36]. This may contribute to obesity prevention as replacing sugary drinks with non-caloric, sweetened drinks reduces energy intake and, subsequently, facilitates weight loss [37]. This is because sugary drinks contain “empty calories”, that is, the energy contained in these drinks is not satiating. Specifically, people do not compensate the calories contained in beverages by eating less, but consume just as much food irrespective of the caloric content of the beverages [38, 39]. Thus, although people eat the same amount of food, those who consume caloric soft drinks consume more calories.

In conclusion, effective obesity prevention approaches may, of course, be inspired by alcohol and tobacco prevention and the food addiction concept may help increase acceptance of such actions in the general public. However, the success of such approaches does not depend on whether people are addicted to food or not. Therefore, it seems unnecessary to invoke an addiction to food (or caloric beverages) to implement promising obesity prevention strategies derived from the addiction field.

## Treatment Implications

Several implications of the food addiction concept for the treatment of eating disorders and obesity have been proposed in the literature. These include suggestions for possible new pharmacological approaches, which have been comprehensively discussed elsewhere [40]. In this article, the focus will be on providing an addiction framework for psychoeducation, psychotherapeutic and other techniques, and abstinence models.

### Psychoeducation

Providing an addiction framework in therapy may reduce perceptions of personal failure. As the large majority of individuals with binge eating-related eating disorders displays an addiction-like eating behavior, this may resonate well with most patients. In patients with BN, for example, it has been reported that using addiction as a treatment metaphor can be helpful to motivate change [41, 42]. For most obese persons, however, providing an addiction explanation in therapy may be inappropriate as only a minority of individuals with obesity displays an addiction-like eating behavior. Similarly, while some evidence suggests that the food addiction concept reduces external stigma and self-blame, it may adversely affect self-efficacy and distract attention away from the significant role of exercise for weight regulation and health [33, 43].

### Psychotherapeutic and Other Techniques

A range of treatment elements have been proposed to be implicated by the food addiction concept, which include acceptance- and mindfulness-based techniques for managing food cravings, cue exposure and response prevention, promoting emotion regulation skills, and motivational interviewing [44, 45]. Besides these techniques, there are numerous contemporary approaches that have been examined for reducing craving and consumption in both the food and substance use domain such as non-invasive brain stimulation, bio-/neurofeedback, cognitive bias modification, and executive function trainings (Table 1).

**Table 1:** Some examples of intervention approaches that have been applied for reducing craving for and consumption of both food and addictive substances

General area	Specific technique	References
Brain stimulation	Transcranial direct current simulation	[46, 47]
	Repetitive transcranial magnetic stimulation	[46–48]
Bio-/neurofeedback	Heart rate variability biofeedback	[49]
	Electroencephalography neurofeedback	[50]
	Functional magnetic resonance imaging neurofeedback	[50]
Cognitive bias modification, executive function training	Attentional bias modification	[51]
	Approach bias modification	[52]
	Motor response inhibition training	[53, 54]
	Working memory training	[55, 56]
Other techniques	Mindfulness-/acceptance-based strategies	[57]
	Ecological momentary intervention	[58]
	Working memory load/interference	[59]
	Cue exposure and response prevention	[60, 61]
	Reappraisal training	[62, 63]

Across different substances (including food), the experience of craving and its cognitive and neural mechanisms are largely similar [59, 64, 65]. Accordingly, interventional strategies that successfully reduce craving for and consumption of alcohol, tobacco, etc. can likely be applied to reduce craving for and consumption of food as well (and vice versa; e.g., [66]). However, this does not require the presence of food addiction. In fact, while the techniques summarized in Table 1 have been tested in clinical samples (e.g., in individuals with BN, BED, or obesity) on the one hand, they have also been applied successfully for reducing food craving and consumption in non-clinical samples (i.e., usually healthy, young, female students) on the other. Thus, it seems that while food-related interventions can, of course, be inspired from the addiction field, such techniques can successfully reduce food craving and consumption without assuming that individuals are addicted to food.

### Abstinence Models

The primary goal of substance use disorder treatment is abstinence, at least for a period of time [67]. Therefore, adopting an addiction perspective on food and eating suggests that those with an addiction-like eating behavior may be best advised to similarly abstain from eating certain foods completely. In fact, such abstinence models have already been discussed decades ago for the treatment of BN [68] and abstinence is advocated by self-help groups such as Overeaters Anonymous [69, 70]. The definition of abstinence in relation to food is not uniform and may refer to avoiding specific foods or to avoiding specific food ingredients. Thus, abstinence in relation to food may not be accurate from a nutritional or neurochemical point of view. For example, individuals who try to avoid eating sugar may still (inadvertently) consume some foods that contain sugar or, at least, other forms of carbohydrates. Nevertheless, individuals struggling with overeating report that applying an abstinence model helped them to control their eating [69, 70].

Although these promising, anecdotal reports exist, abstinence models contrast current practice in cognitive-behavioral therapy. Specifically, cognitive-behavioral therapy aims at reducing dysfunctional dieting in favor of regular eating with flexible and moderate food consumption with no forbidden foods. Thus, it has been argued that abstinence models may be ineffective or—as they encourage dietary restriction—may even be hazardous, particularly in individuals with BN and BED [71]. Indeed, there is experimental evidence that a selective food deprivation increases craving for the avoided foods in vulnerable individuals. For example, when trait chocolate cravers (who had normal weight) were instructed to refrain from eating chocolate-containing foods (but to maintain regular consumption of all other foods), they reported more intense chocolate craving after 2 weeks [72].

When examining the effects of weight-loss interventions in obesity, however, results point in another direction: food cravings tend to decrease during energy-restricting diets [73, 74]. In fact, it seems that this decrease in cravings is selective for the types of food avoided: cravings for high-carbohydrate foods selectively decreased during a low-carbohydrate diet while cravings for fatty foods decreased during a low-fat diet [75]. Thus, abstinence models may not be as harmful as some propose but may even be advantageous to other dieting strategies. In contrast to popular belief, rapid weight loss does not lead to greater weight regain. In fact, larger initial weight loss during energy-restricting diets seems to be associated with better long-term outcomes [76]. Thus, avoiding certain energy-dense foods completely may help obese individuals to lose more weight faster, which may relate to better long-term weight maintenance even when they follow this diet only temporarily (that is, without requiring life-long “abstinence”).

Rigid dietary control (e.g., completely avoiding certain foods) is usually considered a dysfunctional dieting strategy and, indeed, it relates to higher body mass index cross-sectionally [77]. Yet, the causal direction of this relationship needs to be scrutinized. In a recent prospective study, for example, Morin and colleagues [78] experimentally induced cognitive dietary restraint and did not find any adverse impact on food cravings and body weight compared to a control condition. Moreover, it has also been found that the effects of rigid dietary control on food cravings and body weight are attenuated by higher flexible control [77]. Therefore, dietary restraint does not necessarily have to be dysfunctional as long as flexible elements are added. This is in line with a food addiction model of binge eating behavior, which was recently developed by Treasure and colleagues [79•]. While this model recommends to avoid and abstain from certain foods (e.g., foods with a high glycemic index) rather than encouraging an absolute “no dieting approach”, it also suggests to discourage individuals from restricting healthy foods to prevent them from reaching a state of semi-starvation.

In conclusion, while contemporary cognitive-behavioral therapy encourages flexible eating with no forbidden foods, the food addiction concept implies that abstinence (i.e., avoiding certain foods) may be a helpful treatment element for individuals with addiction-like eating behavior (e.g., those with binge eating). While such abstinence models are already applied in self-help groups such as Overeaters Anonymous, little is known about the long-term success (and possible adverse effects) of such a strategy. Vidmar and colleagues [80•] recently examined an addiction model-based weight loss intervention in obese adolescents, which included abstaining from “problem foods”. While weight loss was comparable to a standard weight loss intervention control group, the “abstinence” group had higher retention rates. More of such studies—and preferably randomized controlled trials that directly compare the two approaches—are desperately needed so that the usefulness of each approach can be evaluated properly.

## Conclusions

Answering the question whether food addiction “is real” crucially depends on how researchers define addiction in general and food addiction in particular. Thus, the food vs. eating vs. not-an-addiction debate will not be resolved anytime soon. When accepting the approach of translating the diagnostic criteria of substance use disorders to food and eating (as is done for the YFAS) as an appropriate procedure for the evaluation of the food addiction concept, many individuals with binge eating—particularly those with BN—show an eating behavior that resembles an addictive behavior. Accordingly, incorporating an addiction framework in the treatment of such individuals may be useful. Whether using an abstinence model (i.e., not eating certain foods) as part of such an addiction framework produces better outcomes (i.e., symptom reduction in eating disorders or weight loss in obesity) than using a flexible model (i.e., that there should be no forbidden foods)—as is done in state-of-the-art cognitive-behavioral therapy—needs to be rigorously tested.

Despite these possibly fruitful implications of the food addiction concept, it seems unnecessary to include food addiction as a new substance use disorder in future diagnostic classification systems because of its large overlap with existing diagnostic entities. As only few obese individuals without binge eating show an addiction-like eating behavior, providing an addiction perspective on eating will likely be inappropriate for the majority of obese persons. Finally, to implement obesity prevention approaches or certain treatment elements inspired by the addiction field does not require that obese people are designated as food addicted. Thus, although an addiction perspective resonates well with many patients with eating disorders and obesity, the necessity and appropriateness of some practical implications derived from the food addiction concept need to be critically evaluated.
